# Lifecourse Health Development: Past, Present and Future

**DOI:** 10.1007/s10995-013-1346-2

**Published:** 2013-08-22

**Authors:** Neal Halfon, Kandyce Larson, Michael Lu, Ericka Tullis, Shirley Russ

**Affiliations:** 1 UCLA Center for Healthier Children, Families, and Communities, 10990 Wilshire Blvd, Suite 900, Los Angeles, CA 90024 USA; 2 Department of Pediatrics, David Geffen School of Medicine, UCLA, Los Angeles, CA USA; 3 Department of Health Services, School of Public Health, UCLA, Los Angeles, CA USA; 4 Department of Public Policy, School of Public Affairs, UCLA, Los Angeles, CA USA; 5 Health Resources and Services Administration, U.S. Department of Health and Human Services, Washington, DC USA; 6 American Academy of Pediatrics, Elk Grove, IL USA

**Keywords:** Lifecourse health development, LCHD, Epigenetics, Systems biology, Genomics, Biopsychosocial, DOHaD, Complexity

## Abstract

During the latter half of the twentieth century, an explosion of research elucidated a growing number of causes of disease and contributors to health. Biopsychosocial models that accounted for the wide range of factors influencing health began to replace outmoded and overly simplified biomedical models of disease causation. More recently, models of lifecourse health development (LCHD) have synthesized research from biological, behavioral and social science disciplines, defined health development as a dynamic process that begins before conception and continues throughout the lifespan, and paved the way for the creation of novel strategies aimed at optimization of individual and population health trajectories. As rapid advances in epigenetics and biological systems research continue to inform and refine LCHD models, our healthcare delivery system has struggled to keep pace, and the gulf between knowledge and practice has widened. This paper attempts to chart the evolution of the LCHD framework, and illustrate its potential to transform how the MCH system addresses social, psychological, biological, and genetic influences on health, eliminates health disparities, reduces chronic illness, and contains healthcare costs. The LCHD approach can serve to highlight the foundational importance of MCH, moving it from the margins of national debate to the forefront of healthcare reform efforts. The paper concludes with suggestions for innovations that could accelerate the translation of health development principles into MCH practice.

## Introduction

The last 50 years have witnessed a transformation in our understanding of the causes of disease and contributors to health, yet health policy and healthcare practice have been slow to respond. Until the latter part of the twentieth century, simple biomedical models, closely aligned with the mechanistic thinking of the industrial age, dominated understanding of the genesis of illness. These models drove the first era of health care, which focused on the treatment of acute illness, injury, and infectious diseases [1–3]. As evidence subsequently accrued for the role of social and behavioral contributors to illness, newer bio-psychosocial models influenced the second era of health care, supplementing acute services with programs designed to manage chronic illnesses over longer time-frames, and to change unhealthy lifestyle choices. At the same time, social services expanded to provide supports to improve quality of life for patients living with chronic conditions. Yet health and social services remained largely separate, and there was only limited integration of physical and psychological health programs. Starting in the 1980s, a series of landmark epidemiologic studies by Barker, Wadsworth and others led to the realization that events and experiences in fetal life could influence the course of adult health in mid-life [4–9]. Thought leaders subsequently integrated the new ‘fetal origins,’ then later ‘developmental origins of health and disease (DoHAD)’ research results, with findings from lifecourse sociology and psychology to yield newer lifecourse models of health and disease [10–12]. These lifecourse models indicated that a person’s heath trajectory amounted to more than a combination of her genetic endowment and adult lifestyle choices, and that social, psychological and environmental factors operating early in life could have major impacts on both short- and long-term health outcomes.

Initially criticized for appearing overly deterministic and failing to fully address the complexities of human development [13], lifecourse models have since expanded to include the contributions of multiple risk and protective factors operating throughout the lifespan to the course of health trajectories over time [14]. The lifecourse health development (LCHD) model goes further, examining these influences from a developmental perspective that includes the importance of early relationships, addresses the unique aspects of different life stages (e.g., early childhood, adolescence), and incorporates emerging ideas from biological systems theory [15]. Researchers studying epigenetic mechanisms and systems biology continue to make discoveries that are readily incorporated into rapidly evolving LCHD models [16, 17]. Scientists are discovering plausible biological mechanisms that could account for relationships proposed in these models, e.g. the links between stress in early childhood and cardiovascular disease in mid-life.

Now, at the start of the third era of health care, the overarching goals of the health system will increasingly focus on optimizing population health. Yet the gap between our understanding of the causes of disease and what contributes to the development of health and the actual design and operation of the health care system has widened to a gulf [2]. At a time of intense national debate on the future of health care, maternal and child health finds itself at the margins of the discussion, yet lifecourse models dictate that it should be central to any reform efforts. Addressing the health risks that occur early in life is important not just in terms of improving later adult health, but in setting a strong foundation for the entire nation’s well-being.

In this paper, we trace the evolution of the LCHD model and consider its growing implications for maternal and child health policy and practice. The paper is divided into three sections. The first addresses the past, reviewing the evolution of lifecourse-focused research and the eventual convergence of different research streams into a new, integrated LCHD synthesis. The second considers the present, describing the basic tenets of the existing LCHD model, and discussing the “mismatch” with the design and operation of the existing healthcare system, with a focus on maternal and child health. The final section looks to the future, considering how the LCHD conceptual framework is likely to evolve. We predict that notions of LCHD and modern post-genomic notions of biological system function [18–20] will continue to be informed by new and emerging investigative techniques, eventually uniting into an even more integrated over-arching concept of “health development.” The maternal and child health services of the future will be designed to support the optimal health development of the next generation, potentially transforming individual and population health outcomes.

### The Past: Evolution of Lifecourse Thinking and Emergence of the Lifecourse Health Development Model

**Fig. 1:**
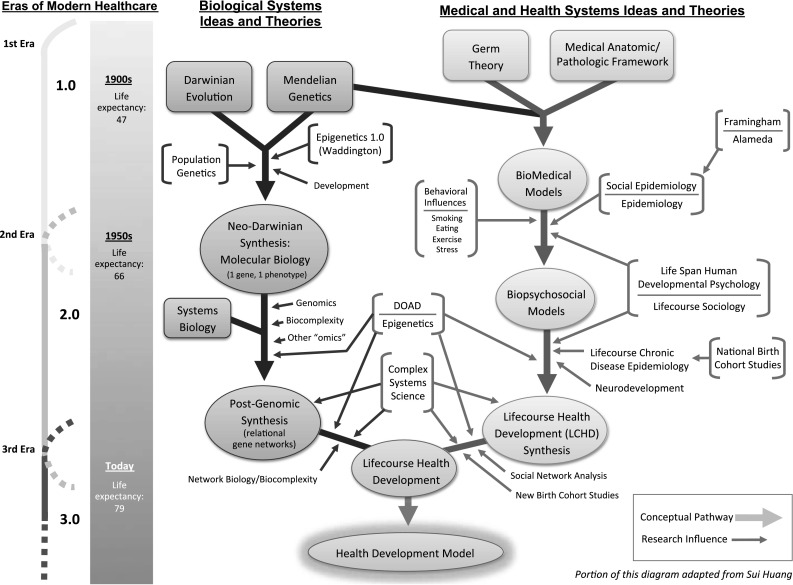
The evolution of health development: this figure diagrams the evolution of two converging and interacting streams of scientific inquiry and conceptual model building. The first stream of Biological System Ideas and Theories charts the development of major conceptual constructs in relation to new ways of understanding how biological systems function. It shows how Darwinian notions of evolution and Mendelian notions of genetics were influenced by other fields of biology but eventually resulted in the Neo-Darwinian synthesis that forms the basis of modern molecular biology. This stream has continued to evolve under the influence of new discoveries in systems biology, genomics, epigenetics, and the application of complex systems science to biological systems. The Medical and Health System Ideas and Theories charts the evolution of the simple, linear and mechanistic biomedical model, and how the biomedical model of health and disease was transformed into a more hierarchical, dynamic and multiply determined biopsychosocial model, which has subsequently evolved into a complex, relational model of LCHD. The Eras of Modern Health Care suggest the approximate timing of these conceptual changes in relationship to how health care has been organized and delivered

Multiple scientific streams have contributed to the development of lifecourse theory. In this section, we consider the principal ideas and theories that have influenced thinking about biological systems on the one hand, and medical and health systems on the other (see Fig. 1). For much of the last century, the development of new conceptual models that explained the function of biological systems proceeded on a parallel but separate track to the constructs underlying the more applied sciences of medicine and public health. A major contribution of the LCHD model is that it serves to integrate these two complementary tracks into a single cohesive framework.

#### Biological Systems Ideas and Theories

Darwin’s Theory of Evolution and Mendel’s notions of discrete genes as the building blocks of heredity converged in the latter part of the twentieth century in a Neo-Darwinian synthesis that has informed our understanding of the basic biology of human development. The study of population genetics, coupled with advances in molecular biology, uncovered the basic scheme of “genes/DNA → mRNA → proteins” that has served as a foundational construct for modern molecular biology. Although the scheme revolutionized understanding of the ways in which genes exerted their effects on biological functions, it led initially to overly deterministic genotype-to-phenotype models that linked single genes to single identifiable phenotypes [21].

Newer findings from the fields of systems biology, genomics and protenomics suggest that an individual’s genetic profile may be less deterministic than once thought. As long ago as 1942, even prior to the discovery of DNA, Waddington coined the term “epigenetics” to describe, in concept, how genes might interact with their surroundings to produce a phenotype. Recent studies have demonstrated that changes in gene expression can result from mechanisms such as changes in DNA methylation and histone methylation rather than changes in the underlying DNA sequence. These same studies are revealing that some of these epigenetic changes are also heritable, suggesting that epigenetic mechanisms provide a route through which environmental exposures can influence the expression and regulation of specific genes, sometimes resulting in permanent changes in phenotype [22, 23]. Studies of epigenetics, gene-environment interactions, and gene–gene interactions have led to a more nuanced understanding of the ways in which genes are regulated and expressed. Gene networks interact both with each other and with the environment in complex, dynamic ways that influence the development and function of biological systems [21, 24]. This new, post-genomic biological synthesis suggests that genetic expression, and the architecture and function of biological systems, may be influenced by both the nature and timing of environmental exposures (see Fig. 1). This new synthesis readily integrates with and informs dynamic models of health development (see below), which posit that environmental exposures and events early in life can influence biological systems in ways that have lifelong effects, and that these varied effects may differ depending on the timing of the exposure in relation to the child’s developmental stage.

#### Medical and Health Systems Ideas and Theory: Germs, Genes and the Biomedical Model

Researchers from basic science, clinical, epidemiologic, social and psychological disciplines have each uniquely contributed to the evolution of different models of disease and health. (See Fig. 1) These models have, in turn, influenced the development of our modern system of health care. Historic studies in the late nineteenth century by Pasteur, Koch and others resulted in the development of germ theory, which proposed that most infectious diseases are caused by microorganisms that invade the host. This discovery paved the way for the development of effective treatments, ushering in the era of modern medicine. Early physicians used an anatomic/pathological approach to classifying diseases based on the localized lesions they empirically observed and measured, first at the macro level and later microscopically. Aligned with the mechanistic ontology of the emerging biosciences of the day, the biomedical model defined disease as the breakdown of body parts and mechanisms, which transformed the body from its “normal” healthy state into one that required “repair.” Consistent with the mechanistic zeitgeist of the industrial age, germ theory and Mendelian genetics provided a way of understanding the mechanisms of disease causation.

While germ theory posited that there was a specific etiology for each disease, the rise of modern genetics, informed by neo-Darwinism, similarly posited that there was, largely, a one-to-one correspondence between a gene and its specific phenotype. These mechanistic, biomedical models drove the first era of modern healthcare, which successfully resulted in the control and treatment of infectious diseases, and in lifesaving surgical procedures focused mostly on mechanical fixes to injured, malformed, or degenerating body parts. An explosion of pharmacological innovations that used new molecules to alter the chemical dynamics of different body systems soon followed. Maternal and Child Health services concentrated on reducing maternal, perinatal and infant mortality, and on treating acute illness and injury, with more emphasis on children “surviving” than “thriving.”

During this first era, life expectancy increased from 47 years in 1900 to 66 years by 1950 in the US, with similar shifts in other industrialized nations. This dramatic increase in longevity was associated with a major epidemiologic shift from the acute and infectious diseases that dominated the first era of healthcare to the growing number of chronic health conditions that would come to define the second “chronic disease” era. Meanwhile, scientific discovery prompted revision of simple models of disease causation to more stochastically-versed multiple risk factor models.

#### Multiple Risks and the Biopsychosocial Model

Transformed by the Framingham study that was launched in the early 1950s, cardiovascular disease became the new prototype of chronic conditions determined by multiple behavioral, social, and biological risk factors. Behavioral factors such as smoking, eating patterns, exercise and stress sparked clinical interest as they appeared potentially mutable and open to interventions. Other longitudinal epidemiologic studies such as the Alameda County study also supported this notion of cumulative disadvantage or risk, whereby complex, interrelated social, psychological, and behavioral factors exerted health impacts not over minutes, hours and days, but over extended time frames of weeks, months, and years [25, 26]. Metaphors like “weathering” were used to describe how exposures to different risks gradually scrape away at the “protective coating” that keeps people healthy [27, 28]. These epidemiologic studies demonstrated that disease was, at least in part, socially patterned with most common health conditions occurring more frequently in individuals of lower socio-economic status. This observation was not new—in fact, Virchow had reported it in the nineteenth Century, but it had had little impact on mainstream medicine. Public health, however, with its population focus, readily incorporated socio-economic factors into interactive models of disease causation. Throughout these developments, maternal and child health remained “on the periphery” of discussions about health in adulthood, with the prevailing wisdom being that much of mid-life disease was the product of genetic predisposition coupled with the effects of adult lifestyle choices.

Using ideas from General Systems Theory, George Engle highlighted the limitations of a strictly biological model that sanctioned the separation of mind and body; instead, he suggested that biological, psychological and social systems not only interrelate, but are interdependent. Echoing concepts by contemporaries including Bronfenbrenner’s ecological conceptualization of human development, and Sameroff’s transactional model of psychological development, Engle’s Biopsychosocial (BPS) Model suggested that illness resulted from dynamic interactions between different body systems and clusters of social systems [29–31]. Despite the general acceptance of the BPS model, Engle’s vision for its impact on clinical practice has never been fully realized. For example, the health care system remains more focused on the diagnosis and treatment of conditions that can be verified by objective testing than on the patient’s subjective experience of ill health, while management of mental health problems remains fragmented, and limited by many insurers.

#### Lifecourse Sociology

Just as health researchers started to realize the importance of social factors in the genesis of health and disease, social scientists were studying how the rapidly changing social circumstances of the second industrial revolution were transforming the developmental pathways of different generations. Elder, Clausen and others championed lifecourse theories that attempted to distinguish how different social pathways were constructed, and how social institutions and historical events shaped the roles, personal experiences, transitions, and trajectories that individuals and groups experienced [32, 33]. Macro-level social processes and social relationships influenced interlocking trajectories at different ages, stages, and transitions of development [34]. Untangling age, period, and cohort effects, and understanding the cumulative impact of experience on socially- and institutionally-constructed life pathways, formed the basis of the emerging field of lifecourse sociology. For example, the experiences of low socioeconomic status, discrimination, and racial segregation could have different effects on health for different cohorts based on compensatory and mediating factors such as the availability of healthcare, or the impact of different social policies [35, 36]. Alwyn suggested five principles that characterized this new lifecourse approach in the social sciences:

1. Lifespan development—human development and aging are lifecourse processes;
2. Agency—individuals construct their lives through choices and actions they take within social structures that provide opportunities and impose constraints, and within historical contexts that do the same;
3. Time and place—lives of individuals are embedded and shaped by historical time and the place where they live;
4. Timing—developmental impacts of events, experiences, and transitions are conditional on their timing in a person’s life;
5. Linked lives—people’s lives are lived interdependently (e.g., husband and wife, siblings).

Health researchers became interested in these principles, considering how they might relate to the development of health and disease.

#### Lifespan Human Developmental Psychology

For more than a century developmental psychologists have attempted to explain how individual differences emerge at different ages and stages [37, 38]. More recent conceptualizations suggest that human development is influenced by endogenous characteristics (i.e., each individual’s adaptability, plasticity, resilience, and reactivity) interacting with exogenous factors (i.e., external physical, social, and psychological environments). As lifespan research has matured, the evidence clearly suggests that these complex and dynamic interactions cause human behavior to continuously change from conception to death [37, 38]. Lifespan human development psychologists focus on the individual’s capacity to adapt to events and experiences [39, 40], i.e. the plasticity associated with individual development (ontogenesis), whereas lifecourse social science researchers emphasize “sociogenesis,” or how life pathways are informed and structured by different socially-constructed developmental scaffolding and constraints. In short, psychologists have focused on how *endogenous* ontogenetic processes influence lifelong developmental trajectories, while sociologists have focused more on *exogenous* factors.

Yet research on “linked lives”—where the common and differential impact of shared exposures are experienced by individuals whose lives are linked (e.g. spouses, workers in a town)—and work on transitions and turning points that are biologically (menarche, menopause) or socially (e.g., transitions from preschool to kindergarten, school to work, work to retirement) determined have each benefited from consideration of both endogenous and exogenous factors. As the sociological approaches to lifecourse, and the psychological approaches to lifespan human development research have converged into a more integrated discipline of developmental science [41–43], conceptual models in the developmental sciences increasingly include relationships that form part of complex adaptive systems [44]. Understanding, measuring and modeling this degree of complexity is presenting new challenges for study design and analysis [44]. Nonetheless, it resonates with advances in basic biological research, where studies are increasingly focused on the ways in which biological systems interact, and on the complex properties of these systems and interactions.

Many researchers and thought leaders have contributed to the conceptual evolution and empirical evidence supporting a more integrated developmental systems theory [37, 41, 45–50], which built upon earlier behavioral and biological theories [44]. Overton and Lerner recently proposed “Relational Development Systems Theory (RDST)” [18, 38, 51, 52], which suggests that a person’s development is embedded in, organized by, and co-regulated by his or her surrounding environment. Developmental regulatory functions are best understood as mutually influential, bi-directional, person–context interactions. RDST sees individuals as active co-developers of their own developmental pathways, adaptively responding to different biological, social, cultural, and physical environmental contexts that they also influence. RDST has been used as a theoretical foundation for research on self-regulation and youth development, and has added a stronger relational dimension to lifecourse thinking.

#### Developmental Origins of Health and Disease and Lifecourse Epidemiology

Pioneering work by Forsdahl, Barker, Wadsworth and others identified influential fetal and early childhood factors, including socio-economic status and birth weight, for a range of adult health outcomes including cardiovascular disease [7, 53–57]. New theories of Fetal Origins, then later Developmental Origins of Health and Disease (DoHAD) were proposed to explain these findings. The “Barker hypothesis” posited that under-nutrition during pregnancy results in a change in “fetal programming” that can permanently shape the developing body’s structure, function and metabolism in ways that predispose to disease decades later in adulthood. Gluckman and others later suggested that, deprived of plentiful nutrients, the fetus makes a “predictive adaptive response,” developing metabolic pathways that would be best suited to a future nutrition-poor environment. After delivery, when nutrition is in plentiful supply, the infant’s metabolism is now mismatched with an environment rich in cheap and plentiful calories, predisposing to the development of metabolic syndrome, relative insulin resistance and obesity [58, 59]. Subsequent rapid catch-up growth after delivery appears to confer even higher risk of adult-onset disease [60]. Developmental origins theories acted as the foundation of early lifecourse models of health, and shifted the time frame of interest for medical studies from months and years to decades and the entire life span [61].

The findings from this burgeoning field of DoHAD research resonated with the previous work of social epidemiologists like Cassel, Syme and Marmot, and health services researchers such as Starfield who had already adopted a more complex, multidimensional “web of causation” set of constructs to explain the onset of disease [57, 62–66]. A growing body of new research described the “embodiment of disease risk” by demonstrating how different social, cultural, and psychological exposures quite literally “get under the skin,” and are encoded or embedded into developing bio-behavioral systems [30, 57, 62, 63]. Later longitudinal cohort studies from Britain, Sweden and New Zealand provided further evidence for the social patterning of early life risks, and their relationship to an expanding number of adult chronic health conditions, including diabetes, chronic lung disease, and depression. This new field of lifecourse chronic disease epidemiology built on the earlier DoHAD work, and prompted researchers to look for mechanisms that could explain these observed relationships [10, 11].

#### Epigenetics and Neurodevelopment

Recently, epigenetic studies have provided clues to the mechanisms that might underlie the process of what has now been termed “biological embedding” [67–72]. These studies demonstrate how gene expression can be modified in response to environmental cues, and that biological and behavioral traits can even be perpetuated across multiple generations. Complementary studies of the developing brain have demonstrated how stress and social adversity are embedded into the biology of human development during sensitive and critical periods [70–72]. Animal models have shown that early experiences of adversity compared with comfort can lead to demonstrably different DNA methylation patterns in neural tissue, and different functional levels of neurotransmission capacity [73–75]. Similar methylation alterations have been demonstrated in children who have experienced adversity associated with maternal stress in the early years [76]. Risky families and toxic environments embed their influence through developing neural, immune and endocrine pathways, resulting in lifelong changes in bio-behavioral function [77–81]. This research on neural development, stress and biological embedding has provided an important empirical and conceptual bridge between observed social gradients in health and the experience-dependent influences on bio-behavioral systems that occur during the process of human development [15, 70].

In several ways, the converging relationship between lifecourse chronic disease epidemiology, neurodevelopmental, and DOHaD research is analogous to the converging relationship between lifecourse sociology and lifespan human development psychology (see Fig. 1). DOHaD and neurodevelopmental research has focused more on individual differences in developmental plasticity from early development through old age (ontogenesis), leading to a growing understanding that epigenetic factors can influence non-germline heredity [82]. In contrast, lifecourse chronic disease epidemiology has focused more on social class, social gradients, and the social scaffolding of exposures (sociogenesis). This conceptual convergence has prompted the inclusion in longitudinal cohort studies of both perspectives, not only measures of phenotype, but of genetic, epigenetic and other bio-behavioral adaptations, [83, 84] and of social environments.

Emerging cross-linkages between these once separate strands of research opened the door for a new conceptual synthesis that could integrate current knowledge from all of these fields of biology, genetics, epigenetics, neurodevelopment, and lifecourse epidemiology.

#### Early Lifecourse Health Development Synthesis

By the year 2000, researchers and other thought leaders began to reconcile prevailing biomedical and biopsychosocial models of disease causation with new ideas about the dynamic role of varying psychological and social factors, the developmental timing of lifecourse influences, and the variable expression of genetic and epigenetic mechanisms. These researchers began to integrate the findings of the emerging fields of DOHaD, lifecourse chronic disease epidemiology, and neurodevelopment into a new set of constructs about human health development [70, 85, 86]. In 2002, building off the initial work of Hertzman and colleagues, Halfon and Hochstein presented a new synthesis of this emerging body of scientific work that they termed the LCHD model. The LCHD model sought to explain how health develops over an individual’s lifetime, and to use this new synthesis to guide innovative approaches to policy development and research. By providing a better understanding of health development, the model sought to focus attention on the impact of risk and protective factors early in the lifespan, and to help shift the emphasis of clinical practice from treatment in the later stages of disease to promotion of more effective prevention and intervention strategies focused on optimizing the development of health [15]. They also argued that this emerging LCHD framework would have profound implications for how health was measured, how health care was organized, and how health systems were financed. By proposing a dynamic transactional model of health development and disease causation, this early LCHD framework largely coalesced around the following principles:

- Health is a developmental capacity of individuals;
- Health development can be represented by health development trajectories;
- Risk factors and protective influences are arrayed in a relational ecological matrix that are dynamically transacting with an individual’s developing biological and behavioral capacities;
- Risk factors and protective influences can have a bigger impact on health development during sensitive and critical developmental periods when biological and behavioral regulatory systems are being initialized, programmed and implemented. Heightened levels of developmental plasticity during these sensitive periods provide for greater mutability and change;
- Risk, protective and health promoting influences can work through different complementary and often interacting mechanisms including:
  - Biological and behavioral embedding during sensitive and critical developmental time periods that can lead to latent effects not clinically observable for years and decades;
  - Cumulative influences over prolonged time frames;
  - Pathways of socially-constructed and culturally-linked factors that provide a type of “social scaffolding” that tends to channel health development toward increasingly predictable outcomes.

By providing a new synthesis of ‘biological system ideas and theories’ and ‘medical and health system ideas and theories,’ the LCHD framework provided a “conceptual bridge” by linking newly emerging results from lifecourse epidemiologic enquiry with the latest findings from bench research in genetics and molecular biology (see Fig. 1). In doing so, the model incorporated an articulation of how, for example, gene-environment interactions and epigenetic mechanisms might explain epidemiologic relationships that had puzzled clinical researchers for decades. This new framing had particular salience for the field of maternal and child health by highlighting the importance of fetal development, early childhood, and the entire “childspan” on how health and disease develop, not just in childhood, but throughout the lifespan. Moreover, the LCHD framework underscored the folly inherent in attempting to improve health in adult life while ignoring influences operating during the early years. In the next section, we will consider how this first articulation of the LCHD model has continued to evolve, and discuss the impact the model has had on Maternal and Child Health.

### The Present: The Lifecourse Health Development Model and its Application to Maternal and Child Health

Since the LCHD model was first synthesized, there has been an explosion of empirical evidence supporting the initial premises of the LCHD framework, and a growing understanding of a range of epigenetic mechanisms that may influence health development [23, 74, 76, 87–90]. These include evidence about neural and endocrine responses to adversity, how evolutionarily adaptive “defensive programming” in utero and early in life may predispose an individual to greater vulnerability to pathogens, and future adversity [60, 71, 91, 92], and how gene-regulatory and transcriptional networks can be induced into self-perpetuating output that render the an individual susceptible to future maladaptive response patterns [93]. At the same time, evidence is emerging that positive influences in the early environment, including attentive caregiving, warmth and nurturing behaviors, coupled with a secure family financial situation can promote more adaptive patterns of neurodevelopment and future positive health. In addition to this research that spans from the epigenetics to the epidemiology of LCHD, there have also been a number of multidisciplinary research papers applying principles of LCHD to major policy issues, including the timing of societal initiatives aimed at optimizing lifelong health and health development [94–96]. As a result, the LCHD model has continued to evolve as these new findings from more advanced epigenetic studies, systems biology, and newer longitudinal birth cohort studies have emerged.

The current LCHD model incorporates this view of health as a dynamic, emergent capacity that develops continuously over the lifespan in a complex, non-linear process.

Today’s LCHD model is best articulated as six basic tenets of health development. In this section, we consider these six basic tenets, explain them in greater depth, and discuss the existing applications of the present LCHD model to maternal and child health.

1. Health is an emergent set of developmental capacities.

Our evolving view of health builds upon the Ottawa Charter’s notion of health as a capacity that enables individual to achieve life’s goals, and the IOM definition of child health as a developmental capacity that gives children the ability “to (a) develop and realize their potential, (b) satisfy their needs, and (c) develop the capacities that allow them to interact successfully with their biological, physical, and social environment” [97]. Presently, health is conceived as an emergent set of capacities of human and other living organisms that develops over the lifecourse as a result of transactions between the organism and its internal and external environments. One of the evolutionary goals of health is to enable the organism to adapt to unknown challenges, and unexpected environments [15, 97–101].

2. Health develops continuously over the life span.

Health develops continuously over the life span, and at any time an individual may be moving toward greater or lesser degrees of health. A person’s health depends on their internal biological and physiologic systems, their external environment and circumstances, and the interactions or relationships between them. Life History Theory suggests that different phases of the life span have evolved into functionally-coherent periods, often categorized as infancy, childhood, juvenile, adolescent, adult, and senescence [102], and that natural selection shapes the timing and duration of these periods to produce the largest possible number of surviving offspring [102]. While Life History Theory has been used to link biological and cultural evolution, and to explore the relationships between evolution and specific life stages as defined by growth and development, it does not account for the capacity of health to promote adaptation, or the process by which health develops. Drawing on the work of Baltes [103], we contend that health development has four distinct functional phases:

- Phase 1—Generativity: The preconception and prenatal period is dedicated to the formation of the organism, and includes the context in which the developing fetus grows. This phase can include the nutritional inputs and neural-hormonal contexts that influence a woman’s reproductive health trajectory, including those early influences on the eggs that are developing in her ovaries years before she is reproductively able [55].
- Phase 2—Acquisition of capacity: The early years of childhood and adolescence through early adulthood are dedicated to the development, acquisition and optimization of specific capacities, including, under optimal conditions, investing in future health potential and anticipated developmental reserves.
- Phase 3—Maintenance of function: The middle years of life comprising adulthood and early middle age are dedicated to maintaining function of these capacities in the face of accumulating risks and ongoing weathering.
- Phase 4—Managing decline: The later years of old age are dedicated to managing, adjusting, and adapting to functional decline of various body and regulatory systems.

There is some overlap between phases. For example, acquisition and optimization of capacities concentrate in the earlier years, but continue for certain types of functionality well into the phase of decline.

Health trajectories are often used to represent the shape, pattern, and slope of these different phases of health development. Given the complexity of human health development, true individual health trajectories can only be constructed in retrospect. Nonetheless, population health trajectories can be used to demonstrate how health capacities develop across different phases or periods of health development, and the role that risk and protective factors play in influencing different trajectories at a population level. Portraying the arc of health development across the life span can also serve a useful purpose in demonstrating the range of different factors that influence the development of different capacities. A great example of how health trajectories can be used to explain the complexity of health development is provided by the Foresight Report on the Development of Mental Capital and Wellbeing [104]. Health trajectories are increasingly used to understand the developmental patterns and natural history of different disease states [105].

**Fig. 2:**
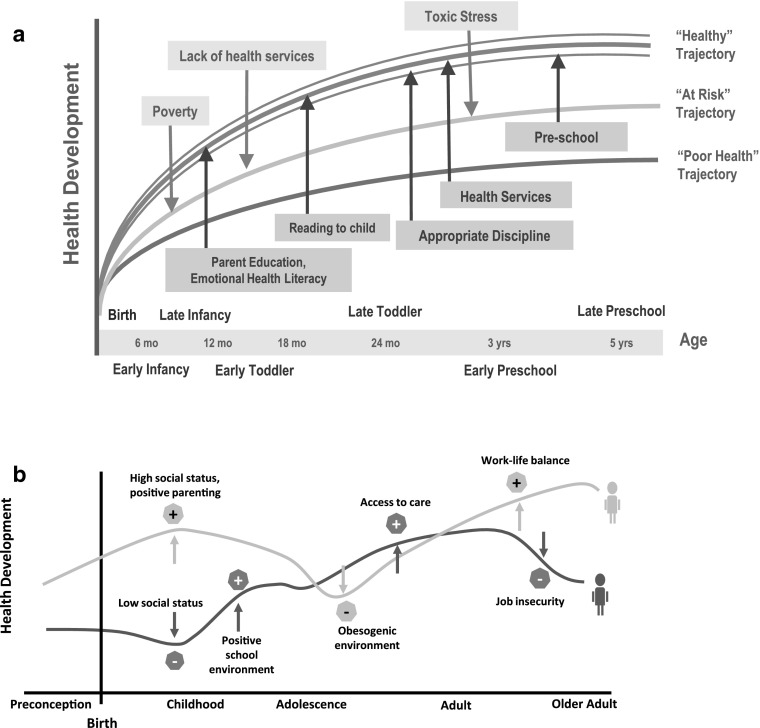
Variable health trajectories: these two figures suggest how health trajectories can be used to illustrate the impact of various risk, promoting and protective factors on health development. In **a**, higher or lower health development trajectories are influenced by the relative number and magnitude of risk and protective factors. **b** Trajectories are not straight, linear, overly determined, or immutable but can be in a constant state of flux relative to different influences at different points in time

Figure 2a illustrates how positive environmental factors, e.g. parent education, reading to a child, and appropriate discipline, can result in a positive shift in an individual’s health trajectory, while negative factors, such as poverty and lack of health services, can shift the trajectory downwards. Figure 2b compares the hypothetical health trajectories of two individuals exposed to a range of environmental influences on health. The figure illustrates the dynamic nature of “health”: One individual starts life with low socio-economic status, but his health improves over time as he is exposed to a positive school environment and quality health care. A second individual starts life in a higher social stratum, but exposure to an obesogenic environment results in his health trajectory falling below that of the first individual by early adulthood. Yet, job insecurity and better work-life balance respectively reverse the trajectories again by late adulthood (see Fig. 2b).

3. Health development is a complex, non-linear process occurring in multiple dimensions, and at multiple levels and phases.

The developmental process that results in the emergence of health cannot be fully understood using a traditional biomedical approach. Attempts to reduce analysis of lifecourse influences on health to simple linear relationships only reveal part of the story. For example, researchers have linked birthweight with cardiovascular health in mid-life using relatively simple linear analyses. Yet birthweight represents only one marker of the individual’s nutritional and metabolic systems that proceed to interact with environmental, social, and cultural systems influencing diet and exercise to result in an adult cardiovascular health system. In turn, readily measurable sentinel events such as strokes and heart attacks represent only partial markers of cardiovascular system function.

Physical, biochemical, psychological, social and cultural dimensions of development dynamically interact to shape the health development process. The processes of health development also occur at multiple interacting levels of organization. Processes at the molecular/genetic level can dynamically interact with each other, as well as with processes at the social and ecological levels, and everywhere in between. Each of these levels can have its own regulatory logic and time signature. For example, the degree to which social and family environmental factors influence gene expression may depend on the strength (dose), timing, and reinforcement of those influences. Several recent studies on the role of adverse social conditions early in life have documented that these early experiences can alter gene expression not only during childhood but in adult life [73, 74, 87, 93, 106–109]. The work of Meaney and colleagues that has demonstrated how different types of maternal grooming behavior can influence gene expression and the development of different synaptic receptors in the brain, resulting in different behavioral profiles, is a good example of this phenomenon [73, 74, 87].

4. Health development is sensitive to the timing and social structuring of environmental exposures and experience.

Three different types of health development pathways have been described in the LCHD literature, classified by Hertzman as latent effects, pathways effects, and cumulative effects [12, 71, 86]. Each process reflects a complex dynamic pathway, and here we modify that typology to distinguish them by either their timing, social construction, or both.

*Time*-*specific pathways refer to the processes of biological embedding that occur during sensitive or critical periods* when developing bio-behavioral systems are most alterable, and when exogenous and endogenous influences can result in different adaptive responses. For example, exposure to specific antigens in utero will program specific immune responses, and exposure to maternal depression during specific developmental phases will lead to alterations in the HPA axis by programming cortisol response patterns.

*Time*-*specific transitions and turning points in health development result from biological, social, and cultural shifts in function, demands, and capacity*. The transition from home to preschool places all kinds of adaptive demands on a young child, including levels of stress and new cognitive, language, and behavioral demands that the child must respond to. Similarly, puberty marks a biological, cultural, and social transition loaded with adaptive challenges, where social and cultural information is being transduced into biological function. Many of these time-specific adaptations are now being linked to epigenetic mechanisms, stimulated by environmental exposure or experience. Higher levels of exposure to disruptive changes and new stresses require that different bio-behavioral systems respond, adapt, and reboot specific routines under different conditions, time demands, and levels of support.

*Time*-*dependent pathways reflect the cumulative influence of different factors that occur over time*, not necessarily in relationship to a time-specific period of heightened sensitivity, and can be additive or multiplicative. For example, the cumulative amount of exercise that an individual engages in will have an impact on their bone metabolism, strength, and long-term risk of osteoporosis. Similarly, sustained levels of inactivity lead to lower levels of physical and cardiovascular fitness. These cumulative effects are not only additive or time-dependent, but can also be time-specific if the exposure overlaps with a particularly sensitive period where the potential for biological embedding is enhanced. For example, more exercise during childhood and adolescence seems to have a protective effect on bone health that can be maintained and reinforced by the cumulative effect of exercise on bone health during later life [110–112]. Metaphors like weathering and burden describe the additive nature of adverse exposures over long time periods. Because cumulative effects can compound over time, ordinary and unremarkable exposures in an impoverished child-rearing environment can result in a heavy burden and a great deal of weather, measured by the loss of health development potential [99].

*Socially*-*structured pathways are those that link experiences and exposures in ways that create recursive,* *iterative, and mutually*-*reinforcing patterns of risk, protection, and promotion*. Socially-structured pathways have both period-specific and time-dependent (cumulative) characteristics. Social and historic contexts shape the scaffolding, supports, and constraints that influence pathways of health development. By arraying risk, protective, and promoting factors into socially-constructed and institutionally-reinforced pathways that interact with bio-behaviorally-sensitive periods of health development, societies can either support growth of positive health development trajectories or reinforce negative ones. The role, relative dose, duration, and interaction of risk, protective, and promoting factors during formative, maintenance, and declining phases of the lifecourse all influence the slope and shape of health trajectories. For example, children growing up in impoverished environments with more risks (e.g., lack of consistent healthcare, exposure to more health risks, higher levels of toxic stress) and fewer protective factors (e.g., high quality preschools, access to appropriate nutritional supports) are more likely to have a lower health trajectory than those children growing up in environments where risks are fewer and protective factors more plentiful and effective. They are also less likely to attend college, and more likely to face periods of unemployment and financial stress in adult life. Unless society alters its infrastructure to provide specific occupational opportunities and supports for the most at-risk youth, risks will continue to multiply over the lifespan, with predictable further declines in health trajectories.

From a clinical perspective, pathways to health and disease may result in the development of clinically recognizable endophenotypes. Endophenotypes represent subclinical disorders or sub optimal transitional health states that are precursors to fully formed phenotypes. The alteration and control of these evolving endophenotypes can emerge from self-organizing gene regulatory networks, from external environmental modification of the epigenetic topology, or from a cascade of multiple gene-environment interactions, representing the influence of the time specific, time dependent and socially constructed influences [21]. For example, the development of metabolic syndrome as a result of prenatal exposures to maternal obesity (time specific), early life exposures to excess calories and limited activity (time dependent and socially structured) represents an endophenotype on the pathway toward emergence of type II diabetes. The clinical identification of endophenotypes creates possibilities for targeted and preemptive interventions aimed at avoiding full-blown disease states. In some cases, endophenotype formation predates onset of overt disease by many years, creating a window of opportunity to shift the health trajectory.

5. Health development is an adaptive process that has been engendered by evolution with strategies to promote resilience and plasticity in the face of changing and often constraining environmental contexts.

Evolutionary forces operating over prolonged time periods have selected for strategies that promote developmental plasticity, or the ability to adapt to a range of environments [17]. This “adaptability,” built into human systems, not only promotes survival of the species in the face of unpredictable changes in surroundings, but promotes behavioral resilience in the face of different types of adversity.

Phenotypic plasticity is the ability of the organism to alter its phenotype in response to environmental challenges, opportunities, barriers, and constraints by immediate adaptive response, altering metabolic demands in order to preserve metabolic capacity and blood flow to vital organs and systems. Immediate responses are distinct from predictive adaptive responses, which are “strategic bets” that the organism makes, based on information received, in this case via the mother’s placenta, to forecast the need to re-program a specific regulatory process to assure future adaptive advantage. The up-regulation of specific metabolic pathways in response to intrauterine nutritional deprivation, including changes in leptin-mediated regulation of carbohydrate metabolism, is an example of such a predictive adaptive process [59, 113–116].

The process of selective optimization, first described by Baltes et al. [46] in 1980 as a behavioral adaptive response strategy, enables the organism to maximize developmental gains and minimize losses. In the face of age-related challenges, and internal and external constraints (e.g., energy, resources, scaffolding, and relationships), the organism must select/choose a process that optimizes some capacities while often limiting others. Facing biological or socially-imposed limits, individuals and specific physiologic systems will begin to invest resources into those processes or behaviors that are deemed physiologically, individually, and/or socially adaptive to new or anticipated constraints. This specialization takes time, energy, and motivation, requiring individuals to disregard other behavioral demands, or physiologic systems to disregard other regulatory processes that are not deemed adaptive to these new developmental limits.

In summary, the process of environmental adaptation is central to the concept of health development. In general, health trajectories rise when biological and behavioral systems are “in synch” with the prevailing environment and fall when there is a “mis-match.” Environmental changes in mid-life pose particular challenges as the individual’s bio-behavioral systems must undergo new adaptations in order to maintain function. Mechanisms of developmental plasticity, which have evolved over a long and varied history of human evolution, allow for adaptive changes to any new environment, with the potential to not only preserve adaptive capacities but to optimize health.

6. Health development is sensitive to the timing and synchronization of molecular, physiological, behavioral, social, and cultural function.

A hallmark of developmental sciences, developmental biology, psychology, and human development has been the important role that the timing of exposures and experiences play in relationship to the functional maturation of developing systems. This has led to notions of sensitive and critical periods, and the role that time specific and time dependent influences play in regulating health development. Time and time frames, despite their importance in setting the cadence of developmental processes, synchronizing the relationships between different subsystems, and defining the units of analysis for period and cohort effects, are often ignored, trivialized, or assumed to be one-dimensional.

The process of health development binds together developmental subsystems which often operate with different time signatures. Genetic modulations happen on a molecular time frame measured in nanoseconds; biochemical modulations occur over milliseconds; homeostatic modulations may take seconds to days to unfold; social norms evolve over years and decades; cultural processes change from years to centuries; and ecological processes normally take millennia. Human biological, social, and cultural evolution has helped to organize how these different systems and levels interact, coordinating these differently-timed regulatory responses so as to optimize the adaptive relationship between humans and their varied environmental contexts [115].

The obesity epidemic provides a good example of the mismatch between different time horizons. Characterized as the end result of too many calories consumed and too few calories expended, the causes of the epidemic have been over-simplified. Human metabolic regulation and control processes evolved in response to a specific ecological environment that existed many millennia in the past, serving our hunter-gather ancestors well as they stored energy between long periods without eating, during an evolutionary past when food was less abundant and human culture had less ability to capture, generate, and produce nutrition [117, 118]. The remarkable capacity to inexpensively produce, distribute, and market calories in the form of fast food products, coupled with profound changes in work, family, and eating behaviors, has created the perfect storm that has influenced the development of childhood obesity. The mismatch between metabolic systems that were selected to function in one historical time period and their ability to function in a vastly different time period is a good example of this kind of disruptive process.

As modern health and healthcare systems have developed and evolved, they have had to adjust time horizons of prevention, treatment, and care (see Table 1). In the first era of modern healthcare, when the focus was on rescuing individuals from the impact of acute and infectious disease, time frame considerations were usually immediate and short term. The second era, focused on stochastic models of cumulative risk and chronic illness, shifted temporal considerations to the longer time horizon of years and decades. As the third era of health begins to embrace and utilize the LCHD framework to understand how health and disease develop, time frames will shift yet again, this time including lifelong and cross generational time frames.

**Table 1:** Healthcare delivery—past, present and future

Healthcare delivery	Health model	Focus	Time frame	Importance of maternal and child health
1.0 Past	Biomedical	Treatment of acute illness and injury	Immediate, short-term-days, weeks	Low
2.0 Present	Biopsychosocial	Management of chronic illness	Medium term-months, years	Moderate
3.0 Future	Health development	Health optimization for all	Lifelong and multi-generational	High

The current and continuously evolving version of the LCHD model aims to promote a better understanding of health as a complex, developmental, and emergent process. The six tenets articulated in this section describe the principles underlying the health development, and suggest potential approaches to improving health trajectories. Together, they provide a framework to explain how multiple factors at the individual (genetics, biome, and behaviors), family, community, social and physical environments as well as policy levels dynamically interact to influence the emergent capacity of health, mediated through the timing and influence of evolutionarily-trained regulatory processes. Individual health pathways and population health trajectories emerge as result of these complex interacting influences and the equally complex biological, cognitive, behavioral and developmental regulatory processes that continuously and dynamically adapt to optimize health function. From a population health perspective, optimizing health development trajectories requires individual behaviors, social strategies and public policies that reduce and minimize the impact of risk and maximize the impact of protective and health promoting factors. Translating this new perspective into health development strategies, and health and healthcare interventions, will be crucial for improving population health and addressing the health impacts of rapid and accelerating demographic, ecologic, and cultural transformations. Briefly, we now highlight some of the impacts the LCHD model has already had on the maternal and child health field.

### Impact of the LCHD Model on Maternal and Child Health

#### Definitions of Health

The idea of health as an emergent, developmental process has implications for the way health is defined and measured. In 2004, the LCHD model helped inform the work of the Institute of Medicine’s Committee on Child Health to propose a new definition of health in their report Children’s Health, the Nation’s Wealth (CHNW) [97]. This definition incorporated the concept of health as a developmental capacity that allows an individual to interact successfully with his biological, physical and social environments. Consequently, measures of health must evolve from a simple focus on the presence or absence of disease to an estimation of levels of functional capacity and health potential—the adaptive capacity to achieve future health goals.

#### Maternal and Child Health Strategic Planning

In 2010, Fine and Kotelchuck [119] applied the lifecourse model as an organizing framework to inform strategic planning for the US Maternal and Child Health Bureau. The authors noted that while MCH public health programs have historically led the way in addressing social and environmental factors that affect health, there has been limited focus on health trajectories across the lifespan, or on continuities from child to adult to old age. Instead, much of MCH public health is currently organized around a “stage of life” approach, with separate programs for women of reproductive age, and for children at different developmental stages. The authors also noted shortfalls in services addressing intergenerational health, and the relationship of parent’s, and even grandparent’s health to children’s health. Lu and Halfon have argued that maternal health plays a powerful role in the persistence of racial/ethnic disparities in birth outcomes, and that solutions will require approaches that cut across generations [120]. Halfon et al. [121, 122] made a strong case for services that focus on optimizing health during critical and sensitive early life developmental stages, and for better integration of health services with social, local government and community-based initiatives. Fine and Kotelchuck [119] similarly recommend a move away from a focus on specific, discrete programs to a more integrated approach to creating a “pipeline for health development” for all children.

#### Research and the National Children’s Study

Researchers have begun to consider the implications of the LCHD model for the maternal and child health research agenda [123]. The model suggests requirements for longitudinal rather than cross-sectional studies, long-term perspectives, data-sets with genetic, physical and mental health, environmental and socio-economic data and the study of positive health states. The US National Children’s Study (NCS) offers an opportunity to address many of these requirements as do most of the other recently launched international birth cohort research efforts.

In the next section, we consider ways in which the LCHD model is likely to continue to evolve, and wider implications for the future practice of maternal and child health.

### The Future: Health Development and the Future of Maternal and Child Health

The LCHD model is not just an incremental improvement on past biomedical or biopsychosocial models of disease causation, but represents a major transition and paradigm shift, with ramifications for how health is measured, how healthcare is organized, delivered, and financed, and what our health system might aspire to achieve. To date, the LCHD model has engendered most interest in those aspects that pertain to “lifecourse” framing and formulation, providing an attractive framework for health and health care researchers that integrates evidence from multiple disciplines into a single model of how health and disease progress across the lifespan. We anticipate that future iterations of the LCHD model will focus increasingly on its “health development” aspects, and on its implications for policy and applications for measurement, health system organization, and MCH practice. Enquiry will also shift from “what caused this disease condition?” to “How can we, given this starting point, improve this individual’s health across multiple domains, and over the short and long term?” This approach will be especially important as the epidemiology of child health continues to shift toward non-communicable chronic health conditions, which will increasingly be understood as health development disorders, and as the goals of pediatric practice move from the relatively narrow focus of treating and preventing disease, to the much broader aim of optimizing each individual’s health development capacity for life. In short, the “LCHD” will continue to evolve into a broader “health development model.”

#### The Science of Health Development

The health development model of the future will be driven in part by the converging frameworks of the health development sciences including epidemiology, epigenetics, DOHaD, developmental psychology, systems biology, and newly emerging fields, such as the analysis of social networks and their contributions to health (see Fig. 1). Here we describe six areas where we expect particularly rapid expansion of research activity and knowledge development.

##### Systems Biology

Genome-wide association studies, designed to isolate and link gene and DNA disease variants, will move toward more gene expression studies focused on gene networks and their phenotypic variants. In turn, full molecular network studies will examine network relationships between DNA variants, RNA, proteins and related metabolites [124]. Moving away from a “single gene-single pathology” model, these studies will likely identify key gene networks that may be involved in a range of described pathologies, involving different organ systems and stages of development [124, 125]. A host of studies on how social adversity modulates DNA transcription with influences on the developing immune, endocrine, and neurological systems are already paving the way in this direction [72, 93].

##### Environmental Epigenetics

Ongoing epigenetic research will continue to elucidate how non-genetic mechanisms can encode stable phenotypes that can respond to environmental contextual changes [126]. Improving measures of environmental influences will require better specificity and quantification of factors like social status, discrimination, prosperity, and stress at the macro level, as well as better measures of cellular and tissue-specific environments that directly regulate networks of gene, protein, and metabolite expression and function, and transform normal physiologic processes into pathologic processes. The identification of environmental factors that act through epigenetic mechanisms to influence the systems dynamics of gene–protein–metabolite regulatory networks noted above will suggest which factors should be targeted through interventions to shift a child’s biological systems toward healthier developmental pathways. Refinements in measuring the environtype/epigenotype will also more precisely define how the epigenetic landscape is changing in relationship to new environmental and evolutionary pressures [72, 127].

##### New Data Cohorts

New longitudinal (preconception and birth) cohort studies will facilitate studies of the epigenetic epidemiology of complex diseases, allowing for the analysis of epigenetic profiles before clinical disease onset [17, 128]. These studies will also employ new measurement tools to better understand how social and family influences during developmental transitions can transform gene expression, alter gene protein networks, and change resultant endophenotypes that will presage future pathology.

##### New Assays and Measures

Population studies of epigenetic variation will need to rely on easily accessible sources of DNA from saliva, buccal smears, and peripheral blood. While these sources may not accurately reflect the local epigenetic variations in target organs and tissues, new techniques to supplement DNA sampling with other metabolic profiles using saliva and peripheral blood are likely to improve measurement precision. New measures of health are also needed to capture not only the pathologic manifestation of health development that has gone awry, but the positive health and health potential that result from optimal health development. The National Children’s Study Health Measurement Network aims to create, test, and apply new multimodal measures and profiles of positive health development. This includes strategies that link measures of biological process (biomarkers), with clinical measures of phenotypic manifestations, as well as self-report of the experience of health or illness. One important challenge is the need to improve measurement of health capacities that are dimensionally consistent across developmental phases, yet sensitive to developmental modulation.

##### New Classification Schemas

Older schemas of disease classification like the International Classification of Disease (ICD) system and the first four versions of the Diagnostic and Statistical Manual (DSM) predominantly rely on a categorical approach that is consistent with simple biomedical models of disease causation. Diseases are classified by body system and spectrum of severity. Dimensional Classification has recently been introduced to supplement the ICD and contribute to DSM-5 to measure functional capacity (International Classification of Function—ICF), reflecting the Chronic Disease Era’s need to evaluate functional capacity in a variety of domains. As the pathways and dynamics of health and disease development become better specified, a dynamic developmental classification system that is informed by the LCHD perspective, and that captures continuity and variation in the development of specific disorders, is likely to emerge.

In summary, accelerated progress on the science of health development holds promise for new opportunities to manipulate environmental factors early in life to enhance the functioning of gene networks and metabolic systems, thereby improving positive health and health potential. At the same time, new health measurement and classification initiatives will result in a greater emphasis on functional developmental health outcomes, rather than on simple categorical descriptions of observed pathologies. Greater use of biomarkers and identification of endophenotypes will facilitate early detection of individuals that are on a pathway to reduced health, allowing for preemptive interventions to avoid full-blown disease states. Taking full advantage of these opportunities, however, will require major changes to our existing health system.

#### Translation of the Lifecourse Health Development Model into Maternal and Child Health Practice

Here we consider some of the major implications for maternal and child health of this new way of thinking about health development over the lifecourse. We highlight those areas where the health development model is poorly aligned with existing policy and practice, and suggest innovations that could accelerate the translation of health development principles into practice.

##### Health Development as a Positive Capacity for Life

The LCHD model moves beyond the traditional clinical focus of diagnosing and treating established illness, and even beyond screening, prevention, health promotion and anticipatory guidance paradigms. In contrast, the goal of health services becomes to achieve, for every individual, a state of positive health that enables her to function at her highest level of capacity to achieve her personal goals. The idea of health development re-frames the conversation between provider and patient (or family, in the case of children) from a negative focus on remediation of deficits to a positive one of moving toward a better state of health both for the short and long term.

##### Early Childhood as a Time for “Intensive Health Development Care”

The LCHD Model is helping us to understand that many health conditions are disorders of development, where adaptive processes have deviated outside of the normal range, or where predictive adaptive responses have resulted in a mismatch between the anticipatory response and the child’s actual environment [129–132] (e.g., the metabolic “up-regulation” that results from intrauterine nutritional deprivation and then predisposes to obesity when postnatal nutrition is abundant; over-activity of the HPA axis in response to high levels of early life stress that predisposes to chronic anxiety). In many cases, maladaptive developmental processes are at work for quite some time before clinically-significant aberrations are recognizable, or the pathway and trajectory of the aberrant developmental process is clinically detectible. As these pathways become better defined through the specification of endophenotypes and the identification of biological and behavioral makers, it will become increasing possible to screen and detect these developing disorders in pre-symptomatic states that may respond to preemptive interventions. The LCHD Model suggests that many of these interventions must focus on early childhood, before critical periods for the setting of biological systems have passed. The identification of a well specified at-risk endophenotype could trigger a multi-faceted response coordinated across health, social services and early education systems aimed at shifting a child’s health trajectory in a positive direction.

Although the past decade has seen significant progress towards integrated early childhood systems of care, too often they remain challenged by incomplete population coverage, lack of identification of sub-optimal bio-behavioral health trajectories, and lengthy delays in initiating interventions. The consequences of these deficiencies will not be fully appreciated until these children reach mid-life.

While the emphasis on early childhood, and the role that toxic stress and other forms of adversity can have on long term bio-behavioral function is now well documented, there is indeed a risk in focusing all of our attention on the earliest years, as if adolescent health care, to paraphrase Paul Wise, becomes something like palliative care [133]. As the research on epigenetic modification of gene expression advances and more is learned about the enormous developmental changes that continue, especially in neurodevelopment, well into an individual’s third decade, any focus on early childhood that precludes other kinds of interventions across the entire child-span leading into adulthood would be unwise and unwarranted.

##### Pre-conception as a New Developmental Stage

While fetal life is now understood to play an important role in childhood and later life health, the importance of the preconception environment into which the early conceptus is implanted has been relatively under-appreciated. This environment is highly dependent on the mother’s health in terms of her genetic make-up, her own past epigenetic influences, her nutritional and metabolic status, any chronic illness, her environmental exposures and her social networks. The stage of the lifecourse between reproductive maturity and conception of the first child straddles adolescent and early adult health services, and is characterized by infrequent attendance at health care encounters and fluctuating insurance coverage. Strong engagement of young people in optimizing their own health development with a view to providing the most positive preconception environment could yield great benefits for the future health of both mothers and children. Failure to address health risks such as substance abuse, chronic anxiety and depression, overweight and obesity, and nutritional deficiencies in youth must be viewed in the context of potential consequences for their future children’s health trajectories, as well as their own health.

##### Identifying Difficulties with Bio-Behavioral Adaptations

A number of the “new morbidities” commonly encountered in child and adolescent health practice might be better conceptualized as difficulties with bio-behavioral adaptation across the lifecourse. Several recent studies have connected environmental and other adversities in pre- and early post-natal life, effects on fetal growth and metabolic dysregulation, and changes in neurodevelopment and stress reactivity with the development of a range of mental health disorders in children and adolescents [130–132]. For example, a child whose early environment has been characterized by poor socio-economic circumstances, prolonged maternal depression, and harsh physical punishments may develop a heightened stress response system that expects an almost permanently hostile environment [134]. Following entry into the school system, he may display either episodes of extreme anxiety, or of aggressive responses to minor social interaction difficulties. Failure to appreciate the child’s early environment, or the nature of his stress response system could lead to inappropriate responses, for example further harsh physical punishment, with continued decline of his mental health trajectory. Instead, management strategies might focus on improving the mother’s mental health, maximizing economic well-being, and even reprogramming his stress response system through cognitive behavior therapy and stress-management strategies.

##### Population Health Development

Several new population-based measurement initiatives are attempting to measure health development of children and the health and development trajectories of geographically-defined populations [127, 135–137]. These data can then be used to inform community-based strategies that focus not only on shifting population health outcome curves, but on shifting the population health trajectory curve [122]. As states are required to establish kindergarten readiness assessments and move to implement new data systems designed to measure the impact of federally-funded home visiting programs, there is a new opportunity for the MCH community to use its considerable expertise with prenatal and early childhood data to establish better measures of health development trajectories. School readiness measurement, using a comprehensive multidimensional measure, can serve as an anchor for a more robust system for measuring health development.

##### Organizing Health Development Systems

Each era of healthcare has had its own version of the health system that has reflected current thinking with respect to logic, ontology, causal models and approaches to health and disease [2, 3]. (See Table 1) In the first era, hospitals and clinic were created to provide rescue care for acute and catastrophic health problems and infectious diseases. Health System 1.0 also developed indemnity health insurance to pay for the unexpected, and to protect families from financial ruin. The second era’s Health System (2.0) recognized the need for management of chronic disease over more extended time frames. Health insurance was redesigned to enable prepaid benefits that would cover anticipated screening and prevention services that targeted a growing number of chronic health conditions. Now that life expectancy has approached 80 years of age and our scientific knowledge is revealing what it takes to enable an individual to live a healthy life into their eighth or ninth decade, the goals of the health system must shift to the complex, lifelong process of optimizing health. Enabled by a lifecourse framework for understanding the intricate process of health development, the new 3.0 Health System will focus on lifelong and cross-generational time frames, and will require new ways of investing in health development and organizing the care system.

##### Investments in Health Development

The LCHD approach urges us to rethink how best to leverage healthcare expenditures, especially during early sensitive periods where health investments are likely to result in compounded gains in health potential and health reserves. While most health economists classify healthcare expenditures as consumption, from an LCHD perspective, some healthcare expenditures are really investments in the individual’s health capital that will build long-term health reserves [15, 138, 139]. Dollars expended in achieving positive shifts in early developmental health will yield dollars saved in mid-life health care costs, with potential for improved work productivity, economic growth, and linked positive health effects for mothers and children and across family members.

Not only do we need to reconsider how traditional health care expenditure can contribute to our overall investments in health development capital, but we also need to consider the full range of social and education investments that provide the developmental scaffolding that enhance a child’s health development potential. The recent IOM report US Health in International Perspective: Shorter Lives, Poorer Health attempts to understand why the US is the sickest of wealthy nations [140]. Echoing LCHD evidence, the report suggests that many poor adult health outcomes can be traced to childhood, including higher levels of childhood adversity and lower levels of childhood expenditures not just on health, but on the social scaffolding that addresses the upstream determinants of health. Countries with better adult health outcomes also have better child health outcomes, reflecting earlier societal-wide investments in health development trajectories.

##### Enhanced Horizontal Integration

Similarly, optimizing lifelong health trajectories is not solely or even primarily dependent on the medical or health sector. Many other influences and inputs are important contributors to an individual’s long-term health capital. For example, optimizing children’s health over the first 8 years of life as a springboard for later health development is not only determined by what goes on in a pediatrician’s office and whether the child is screened for any of a number of early risks. It also depends on the availability of appropriate nutrition, the ability to exercise and play, exposure to rich and rewarding language environments, and having parents who are educated, skilled, and available to guide, supervise, coach, and direct their children down a health-optimizing pathway. Because multiple factors influence health development, successful health optimization will require not only vertically-integrated medical services based on severity and need, but horizontally-integrated health, education, and social advancement services that promote health in all policies, places, and activities [15, 121, 122].

Better horizontal integration of medical care with other non-health services and sectors is a major challenge to the redesign of primary care. Providers must create new networks of connections between both traditional and non-traditional partners to support the full range of necessary prevention, promotion, and optimization activities. Rather than attempting to address childhood obesity primarily in the pediatrician’s office, it becomes more effective and efficient to move the nexus of prevention and preemptive intervention to the school, day care center, parks and recreation sites, and WIC sites. Such approaches require more collaborative, networked models of care that not only integrate physical and behavioral health, but coordinate with social, community and education assets and resources.

Interestingly, this realization of the need to form health-promoting networks is coming at a time when technological advancements have facilitated social and professional networking in ways that would not have been possible even a decade ago. While healthcare providers have been slow to utilize the networking potential of the internet, there is nonetheless a sense that these new technologies have major potential to enhance relationships between patients, clinicians, and researchers, transforming patients into co-developers of their own health care plans, and providing real time monitoring, linking, and communication between all parties involved in the caregiving process. A promising example of such an approach is the Collaborative Chronic Care Network that has been developed to transform the care of children with inflammatory bowel disease (see http://c3nproject.org/).

##### Enhanced Longitudinal Integration

While the current healthcare system attempts to provide some level of continuity of care, there will be many ongoing challenges to providing the kind of longitudinal integration that promoting optimal health development requires, including the fact that reimbursement strategies focus largely on episodes of care. Given the amount of churning in the healthcare marketplace, and current business and financial models, there are very few incentives for health plans, whose enrollees are often members for only a few years, to organize and approach care in a way that is responsive to lifelong preventive and preemptive strategies. Longitudinal integration of health services goes beyond continuity of care with a specific provider, and facilitates anticipatory, early, and preemptive interventions that are designed to build health capital, improve health development trajectories, and avoid future threats to optimal health outcomes.

The Affordable Care Act (ACA) offers the potential for services such as Medicaid to become more longitudinally focused. As a result of the ACA’s expansion of Medicaid coverage to low income adults, and given the fact that because of low social mobility, 40 percent of children born into the lowest income quintile will remain in that low income group for life, a substantial proportion of low income children are likely to be covered by Medicaid for life. This rather significant change in health care coverage policy may provide a new and persuasive rationale for making early life investments that will save on later life health expenditures [141]. This example suggest how policy initiatives such as the Affordable Care Act, together with other forms of social services, Social Security, Medicaid and Medicare, can provide the type of “social scaffolding” that could support an upward shift in health trajectories at the population level. The challenge for those minding and managing the implementation of the ACA is how to use its significant disruptive potential to put in place the kind of horizontal and vertical scaffolding that children need to achieve optimal health trajectories.

## Conclusion: Moving Forward

The LCHD model represents a synthesis of ideas developed over the past few decades to incorporate rapidly emerging evidence on the biological, physical, social, and cultural contributors to the development of health and disease. This framework will continue to evolve as rapidly advancing and converging fields of scientific inquiry connect molecular alterations in development with societal changes and influences. We have attempted to chart how different fields of empirical research and different models of inquiry have facilitated the emergence of this new framework. The six tenets of LCHD that we have enumerated are also in a state of evolution, and will continue to morph as the science progresses. While these tenets begin to outline the contours of a new and emerging paradigm, we consider them as a network of interrelated ideas that will continue to interact and inform each other.

We have also suggested how the LCHD framework can guide the emerging third era of healthcare, and inform a new approach to MCH programs, policy and practice. By highlighting the importance of the early years of life, the LCHD model suggests that investments in the health of the MCH population are likely to yield the greatest long-term health benefits [138, 139, 142]. It also suggests that optimal LCHD occurs when we take a whole child, whole family, and whole community approach. Whole child means promoting the development of the diverse and interdependent capabilities of the whole child, by starting early, providing the comprehensive and integrated health promoting scaffolding that can protect children from harm, minimize risk, and optimize health development [143]. Whole family means supporting the optimal development of parents, and the interdependent capabilities they need to create the relational environment that every child needs to thrive. Given that many families are squeezed for time, lack financial and other personal resources, and do not have the child rearing knowledge, skills, relationships and supports they need, a strategic response requires consideration of new and innovative family support services, centers, and community based programs. Whole community means that MCH uses LCHD informed strategies to synergize polices across different sectors, align traditional silos, integrate services across sectors, and network all community providers in service of optimizing health development trajectories [144]. This places Maternal and Child Health at the center of a high intensity health development system. MCH programs, policies and practices have the potential to play a major role in both generativity and capacity-building phases of health development. Since these early phases are crucial in the genesis of both health disparities and long-term population health and disease burdens, they provide an important opportunity to leverage resources in service of achieving key health policy goals.

Looking toward the future, we recognize that early lifecourse models have given way to less deterministic frameworks that view health development as a dynamic process that continues throughout the lifespan. The third era of healthcare will have as its focus the optimization of health for all. The concept of “health development” should drive policy and practice. Maternal and Child Health, often viewed as “at the margins” of first- and even second-era care, needs to assume a central position as foundational to the optimization of lifelong health. Creation of a health development system that supports connections between social, psychological, biological, and genetic contributors to health will be key to eliminating health disparities [145], reducing chronic illness, and containing healthcare costs. MCH researchers, policymakers, and providers need to assume new leadership roles in creating such a system based on a rapidly-evolving evidence base, and in developing new partnerships that reflect the complex, multifaceted nature of health contributors suggested by the LCHD framework. Such a system has the potential to transform the care delivered to mothers and children, setting in train optimal health trajectories with benefits that not only improve child health outcomes but are compounded through to the end of the lifespan, and even to future generations.
